# The Efficacy of Intradermal Injection of Botulinum Toxin Type-A on Painful Diabetic Neuropathy: A Systematic Review

**DOI:** 10.5812/aapm-136260

**Published:** 2023-10-09

**Authors:** Masume Bayat, Seyed Ahmad Raeissadat, Fatemeh Hojjati, Parastoo Faghani, Nima Naseri, Vahid Ghafari

**Affiliations:** 1 Physical Medicine and Rehabilitation Research Center, School of Medicine, Shahid Beheshti University of Medical Sciences, Tehran, Iran; 2 Faculty of Nursing and Midwifery, Tehran University of Medical Science, Tehran, Iran; 3 Department of Biochemistry, School of Medicine, Hamedan University of Medical Sciences, Hamedan, Iran

**Keywords:** Botulinum Toxins, Botulinum Toxin Type-A, Diabetic Neuropathies

## Abstract

**Context:**

Diabetes is one of the most common causes of neuropathy. Morbidity and mortality increase in patients suffering from diabetic polyneuropathy and are experienced by approximately 10 to 54% of diabetic patients. Severe pain, loss of sensation, increased risk of ulceration, and even amputation are the complications of diabetic neuropathy. Intradermal injection of botulinum toxin type-A (BTX-A) is a relatively novel method for the treatment of painful diabetic neuropathy. This method is becoming popular considering its acceptable and long-lasting pain control and minimal systemic side effects.

**Methods:**

This narrative systematic review aimed to evaluate the effectiveness of intradermal BTX-A injection on painful diabetic neuropathy. The queried databases included PubMed, EMBASE, Cochrane Central Register of Controlled Trials (CENTRAL), ClinicalTrials.gov, Web of Science, Scopus, and Google Scholar. The final search was performed in February 2022, and no time limits were set for the search. All the relevant clinical trials were included. The inclusion criteria and search strategy were set as follows: Type of study: Randomized clinical trial (RCT) or other types of interventional studies; publication date: All published studies until February 22, 2022; sample size: No restrictions; outcomes: Effect on diabetic neuropathy pain; quality: Earning a minimum acceptable score based on critical appraisal; and language: English. The searches and article screening were performed by two independent reviewers to minimize the possibility of bias. In case of disagreement about a study, the comments of an expert (as a third person) were used to resolve the ambiguity.

**Results:**

In a review of 4 RCTs and 1 case-control study on the effectiveness of BTX-A in reducing the pain of diabetic neuropathy, 273 patients were evaluated in total. The lowest and highest number of subjects was 18 and 141. The sex distribution included 43.22% men and 56.77% women, all of whom were 47.8 to 74.8 years old. Three studies were conducted in Iran, Taiwan, and Egypt. The results of this review showed significant improvement in pain reduction, e.g., based on the Visual Analog Scale (VAS) and Neuropathic Pain Scale (NPS). A few studies evaluated sleep and psychosocial complications, and their results indicated a statistically significant improvement in the Pittsburgh sleep quality index (PSQI) and the physical subscale of the 36-Item Short Form Survey (SF-36).

**Conclusions:**

The results of this systematic review demonstrated that intradermal injection of BTX-A causes significant and long-term (up to 12 weeks) improvement in diabetic neuropathy pain. The improvement in sleep and mental or physical functions was not consistent, and no conclusive result could be reached.

## 1. Context

Diabetes is a metabolic disease attributed to the dysfunction of insulin receptors, which reduces insulin levels. Various genetic and environmental factors contribute to the development of this disease. Nowadays, diabetes is one of the most common endocrine disorders that affects approximately 375 million people worldwide (1). This metabolic disease is associated with macro- and microvascular complications. Macrovascular complications cause stroke, coronary artery, and peripheral vascular disease, while microvascular complications culminate in retinopathy, nephropathy, and neuropathy (2).

As noted before, diabetes is one of the most common causes of neuropathy, observed in approximately 10 to 54% of diabetic patients. Diabetic polyneuropathy can cause severe morbidity and even mortality (3). Severe pain, loss of sensation, and a higher risk of ulceration and amputation are the complications of diabetic neuropathy. The primary classification of diabetic neuropathy includes sensory, motor, and autonomic neuropathy. Patients may exhibit only one type or all three types of neuropathy. The most common form of diabetic neuropathy is distal symmetrical polyneuropathy. Diabetic neuropathy can manifest as movement disorder, silent cardiac ischemia, postural hypotension, vasomotor disorder, increased sweating, bladder dysfunction, and sexual dysfunction (4).

Pain, paranesthesia, hypoesthesia, hyperalgesia, and allodynia comprise the clinical manifestations of diabetic polyneuropathy. Tricyclic antidepressants, carbamazepine, gabapentin, narcotics, duloxetine, and pregabalin are some medications used to decrease pain. Besides side effects such as drowsiness, nausea, and seizure, none of these drugs provide complete and long-lasting pain control, either alone or in combination (5).

Considering the above-mentioned issues, there has always been a demand for more effective cure procedures. Recently, botulinum toxin type-A (BTX-A) has been gaining widespread use in different aspects of medicine. Intradermal BTX-A injection is a relatively novel method for the treatment of painful diabetic neuropathy. This method is becoming popular with due attention to acceptable and long-lasting pain control and minimal systemic side effects (6).

Botulinum toxin type-A is a potent neurotoxin commonly used to treat dystonia, muscle hyperactivity, and spasticity. Although the mechanism of action has not been fully elucidated, its pain-alleviating effects on diabetic neuropathy have been revealed in several studies (7-10). Existing evidence supports the hypothesis that the analgesic effect of BTX-A differs from its paralytic effects on muscles. As for the effects of BTX-A on cholinergic transmission in somatic and autonomic nervous systems, studies suggest that BTX-A can control the cholinergic effects of nociceptive and anti-nociceptive systems, which is completely unrelated to its effects on muscles. Moreover, its analgesic effects are enhanced by modulating inflammation, as well as ascending and descending signal transmissions at axonal, ganglionic, and spinal levels (5, 11).

Botulinum toxin type-A acts on presynaptic vesicles by inhibiting the release of neurotransmitters such as acetylcholine and nociceptive neuropeptides, substance P, calcitonin gene-related peptide, and glutamate (12). It also inhibits the expression of vanilloid receptor TRPV1 on the surface of peripheral nociceptors, which is responsible for inflammatory hyperalgesia (12, 13). Considering the novelty of BTX-A application in the treatment of painful diabetic neuropathy and controversies about its effects, this systematic review was designed to evaluate the efficacy of intradermal BTX-A injection on the pain and quality of life of patients with diabetic neuropathy.

## 2. Methods

This was a systematic review to evaluate the effectiveness of intradermal BTX-A injection on pain in diabetic neuropathy. No time limits were set for the search, and every relevant trial published by February 2022 was included.

The databases that were queried included PubMed, EMBASE, Cochrane Central Register of Controlled Trials (CENTRAL), ClinicalTrials.gov, Web of Science, Scopus, and Google Scholar.

Three searches were conducted in the mentioned scientific databases. The initial search was on June 9, 2021, and the last one was on February 22, 2022. All published studies were extracted with the following keyword combinations.

Query: "Botulinum Toxins" [MeSH Terms] OR "Botulinum Toxins, Type A" [MeSH Terms] # AND Diabetic Neuropathies "[MeSH Terms]

The problem/population, intervention, comparison, outcome (PICO) design of the review was as follows:

- Population: Patients with diabetic neuropathy

- Intervention: Intradermal BTX-A injection

- Comparison: Self-controlled patients

- Outcome: Pain reduction, improvement in sleep, and quality of life

The primary outcome was the pain intensity index (Visual Snalog Scale [VAS]), and the secondary outcomes consisted of the Pittsburgh sleep quality index (PSQI) and the 36-item short form survey (SF-36).

The title and abstract of the studies were examined for compliance with the inclusion criteria. All interventional studies that reported the effectiveness of intradermal injection of BTX-A in patients with diabetic neuropathy (based on study criteria) were selected.

The inclusion criteria and search strategy were as follows:

- Type of study: Randomized clinical trials (RCTs) or interventional studies

- Publication date: All published studies until February 22, 2022

- Sample size: No restrictions

- Outcomes: Effect on diabetic neuropathy pain

- Quality: Earning a minimum acceptable score based on critical appraisal

- Language: English

The full texts of the selected studies were assessed in terms of study design and result-reporting quality based on the checklist for critical appraisal of clinical trials (Consolidated Standards of Reporting Trials [CONSORT]). The standard PEDro Scale was used to evaluate the internal and external validity of the final included studies. The scale comprises 11 questions (which evaluate the study in areas of random sampling) that randomize the allocation of subjects to different groups, blinding, and a complete report of statistical analysis and results, adding up to a total score of 11. Studies with a final score of 0 to 3 are defined as low quality, 4 to 5 as relatively good quality, 6 to 8 as good quality, and greater than 9 (out of 11) as very good or excellent quality.

At the final stage, the required information, including the demographic characteristics of participants and findings of the eligible studies, were extracted and recorded in Microsoft Excel. To minimize the possibility of bias, the searches and article screening were performed by two independent reviewers. In case of disagreements regarding the inclusion of some studies, the opinion of an expert (as a third person) was used to resolve any ambiguities. Ethical considerations were respected in all stages of the study.

## 3. Results

**Figure 1.**
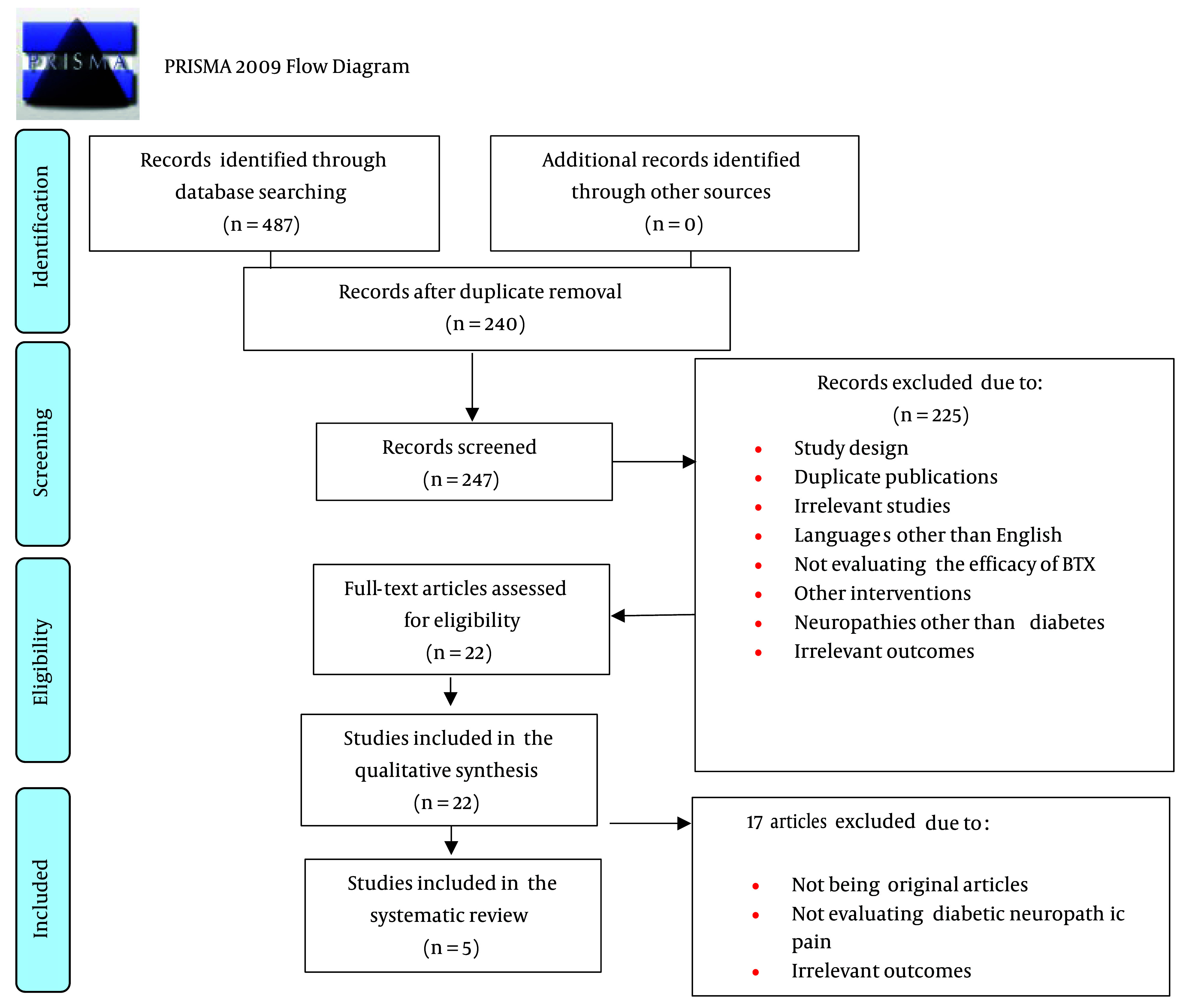
PRISMA flow diagram (14).

A total of 487 records (articles) were gained from the primary search in databases. Out of these initial input studies, 23 were from Medline (PubMed), 20 from Cochrane Library, 191 from Embase, 52 from the Web of Science, and 201 from Scopus. After removing duplicates, 240 studies were excluded, leaving 247 in the screening stage. On an initial screening of titles and abstracts, 225 studies were excluded due to non-compliance with the criteria of the study. These studies included case reports, case series, letters to the editor, correspondence, editorials, opinions, and reviews of animal model studies. In the next screening stage (second screening) performed on full texts, 22 studies were reviewed, and 5 were selected for the systematic review (Figure 1). The characteristics of these studies are presented in Table 1. 

**Table 1.** Characteristics of the Included Studies

Study	Country	Intervention	Sample Size	Follow-up (Weeks)	Outcome Measure	Appraisal Score
**Yuan et al. (** 15 **)**	Taiwan	50 U Botox vs. 0.9% NS	18	12	VAS; PSQI; SF-36	10
**Egila et al. (** 16 **)**	Egypt	50 U Botox vs. 0.9% NS	42	12	VAS; PSQI	7
**Taheri et al. (** 17 **)**	Iran	150 U Dysport vs. 0.9% NS	141	4	VAS; NPS	10
**Ghasemi et al. (** 7 **)**	Iran	100 U Dysport vs. 0.9% NS	40	3	VAS; NPS	10
**Salehi et al. (** 18 **)**	Iran	100 U Dysport vs. 0.9% NS	32	12	VAS; NPS; PSQI; SF-36	10

Abbreviations: VAS, Visual Analog Scale; PSQI, Pittsburgh sleep quality index; SF-36, 36-item short form survey; NPS, Neuropathic Pain Scale; NS, normal saline.

A total of 273 diabetic patients were evaluated in the 5 RCTs on the effectiveness of BTX-A in reducing the pain of diabetic neuropathy. The least and the highest number of samples were 18 and 141 cases. In terms of sex distribution, 43.22% of the subjects were male, and 56.77% were female, in the age range of 47.8-74.8 years. Three studies were conducted in Iran, Taiwan, and Egypt.

The only applied intervention was an intradermal injection of BTX-A vs. 9% normal saline in the dorsum of the foot. The output of the results was divided into 3 general categories: Pain reduction (VAS, NPS), sleep quality improvement (PSQI), reported in 3 studies, and psychosocial function enhancement (SF36), reported in 2 studies.

### 3.1. Pain Reduction

The overall changes in pain are presented in Table 2. 

**Table 2.** Pain Score Changes in the Included Studies

Study	Outcome Measure	Study Design	Intervention Group Pain Score	Control Group Pain Score	P-Value
**Yuan et al. (** 15 **)**	Visual Analog Scale (VAS)	Randomized clinical trial (RCT)	Pre: 6.42 (± 1.11)	Pre: 5.97 (± 1.51)	0.024
			Post: -2.53 (± 2.48) ^a^	Post: -0.53 (± 1.57) ^a^	
**Egila et al. (** 16 **)**	VAS	Case-control	Pre: 8.73 (± 0.46)	Pre: 8.45 (± 0.60)	0.000
			Post: 5.14 (± 1.08)	Post: 6.40 (± 0.60)	
**Taheri et al. (** 17 **)**	VAS, Neuropathic Pain Scale (NPS)	RCT	Pre: 6.7 (± 1.2)	Pre: 7 (± 1.1)	0.001
			Post: 2.1 (± 1.2)	Post: 5 (± 1.5)	
**Ghasemi et al. (** 7 **)**	VAS; NPS	RCT	35% pain reduction after the intervention	Data not provided	-
**Salehi et al. (** 18 **)**	VAS	RCT	_	_	0.001

^a^ Change in VAS.

In the study by Yuan et al., the effect of the injection of 50 units of BTX-A (BOTOX) into the dorsum of the foot over 12 weeks was evaluated in 18 patients. VAS was calculated in the 12th week; it was decreased by 2.53 (± 2.48) in the intervention group and by 0.53 (± 1.57) in the control group (normal saline). The intervention group showed a significant decrease in VAS during this period compared with the control group (P = 0.024). In the intervention group, 44.4% experienced a good response (decrease in VAS ≥ 3) after 12 weeks, while in the control group, none of the patients had such a decrease in VAS score during this period (15).

Egila et al. administered 50 units of BTX-A (BOTOX) into the dorsum of the foot of 42 patients. The VAS value before the intervention was 8.73 (± 0.46) in the intervention group and 8.45 (± 0.60) in the control group. After 12 weeks, VAS values decreased from 8.73 (± 0.46) to 5.14 (± 1.08) in the intervention group and from 8.45 (± 0.60) to 6.40 (± 0.60) in the control group, being significant only in the intervention group (16).

In the study by Taheri et al. on 143 diabetic patients, 150 units of BTX-A (Dysport) were administered over 4 weeks, and VAS scores were evaluated before and after the intervention or placebo. After 4 weeks, VAS values decreased from 6.7 (± 1.2) to 2.1 (± 1.2) in the intervention group and from 7 (± 1.1) to 5 (± 1.5) in the control group, indicative of a significant improvement only in the intervention group. Some NPS parameters, such as pain intensity, sharp sensation, hot sensation, sensitivity, unpleasant sensation, and deep pain, had a significant improvement after BTX-A injection compared with the placebo (normal saline) (P < 0.001 for all). The only difference between the D1 and D2 groups was the feeling of heat, which was more reduced in the D1 group than in the D2 group (P = 0.003). Regarding the other parameters, there was no significant difference between the two intervention groups. Moreover, comparing D2 and N groups, there was no significant difference in dull, cold, and surface pain (P = 0.22, 0.736, 0.069). Comparing D1 and N groups, there was no significant difference in dull and cold sensations. In summary, dull and cold sensations did not improve in the different groups. Moreover, sharp pain decreased more than the other factors after botulinum toxin injection (respectively 80% and 81% reduction in D1 and D2 groups compared to 37% in the placebo group [N]) (17).

Ghasemi et al. investigated the effect of injecting 100 units of BTX-A (Dysport) in the dorsum of the foot of 40 patients over 3 weeks. The numerical value of VAS decreased by 35% (in 7 out of 20 people) after the intervention (absolute VAS scores were not mentioned). Furthermore, the numerical value of all NPS parameters significantly improved after the intervention (7).

### 3.2. Sleep Quality Improvement

In the study by Yuan et al., the results of the pre-intervention CPSQI (Chinese Pittsburgh Sleep Quality Index) showed low sleep quality (CPSQI ≥ 7) in most participants. Although sleep quality improved in the fourth week post-intervention, this improvement was not persistent (15). Egila et al. found no statistically significant improvement in sleep quality in their study (16).

In the research conducted by Salehi et al., PSQI significantly improved after the intervention (12 weeks; P < 0.001) (18).

### 3.3. Psychophysical Function

Yuan et al. assessed the physical and psychological components of the SF-36 quality of life questionnaire. The results demonstrated no significant improvement in this scale (15).

Salehi et al. investigated the psychological and physical components of the SF-36 quality of life questionnaire. No significant change was observed in the psychological component of this questionnaire at the end of the study. However, a significant improvement was seen in the physical component of the scale (P < 0.001) (18).

## 4. Discussion

Diabetic polyneuropathy can cause pain and, subsequently, impairments in the physical and psychological function of patients. According to the results of recent studies, BTX-A injection may provide significant and long-term pain relief.

In all of the 5 reviewed studies, the modifications in pain sensation were evaluated by the VAS index. The NPS index was also used in 3 studies. Sleep quality was evaluated in 3 studies through the PSQI scale. Two studies evaluated the quality of life via the SF-36 scale.

Regarding perceived pain, the results of all 5 studies showed significant improvements after BTX-A injection. In the study by Taheri et al., the injection of BTX-A in one leg had similar pain reduction effects to injection in both legs (17). Studies in mice have also shown that intradermal injection of BTX-A in one leg improves allodynia in both legs, which may indicate the central analgesic effects of BTX-A (1, 3, 5, 6, 19). According to the results of the reviewed studies, BTX-A injection not only resulted in long-term pain reduction but was also well-tolerated by patients and showed no adverse effects (7, 15-18, 20-22).

Regarding sleep quality, which was evaluated in 3 studies, only Salehi et al. demonstrated significant improvements, beginning from the fourth week after BTX-A injection and lasting to the end of the study (18). In the study by Yuan et al., sleep quality improved significantly only in the fourth week after injection (15). In contrast, Egila et al. found no significant improvement in sleep quality with the intervention. The authors discussed that this type of treatment improves only some modalities of pain, and there may be some other types of pain that may be masked during the day and become more prominent at night, interfering with sleep (16). Different results regarding sleep improvement in the studies by Salehi et al. (18) and the other 2 studies (Yuan et al. (15), Egila et al. (16)) may also lie in the difference between the brand of BTX-A injected (100 units of Dysport vs. 50 units of BOTOX).

Only 2 studies evaluated psychophysical function. Yuan et al. observed no significant change (15). In the study conducted by Salehi et al., only the physical components of SF-36 showed significant improvement (18).

Overall, considering long-term effects, relatively low cost, and limited side effects, BTX-A can serve as a treatment modality for patients with long-lasting resistant diabetic polyneuropathies.

### 4.1. Study Limitations

A limitation of this study was the inadequacy of the number of well-designed clinical trials on this subject. Incomplete reporting of pain, sleep, and quality of life indices in the reviewed studies, as well as the use of different measurement tools, made it infeasible to perform a meta-analysis. Another limitation of this study was the use of two brands of BTX-A (BOTOX and Dysport) in different studies.

### 4.2. Conclusions

The findings of this systematic review indicated that BTX-A injection in patients with painful diabetic neuropathy can result in significant and long-term pain improvement. However, results on sleep quality and mental and physical functions were not consistent across the studies.

## Data Availability

The protocols for the research and the associated data set used and/or analyzed during this study are available from the corresponding author upon reasonable request.
